# Supplementary material for: Disentangling the effects of corticotrophin releasing factor and GABA release from the bed nucleus of the stria terminalis on ethanol self-administration in mice
Source: Neuropsychopharmacology. 2025 Sep 6;50(13):2040–50. doi: 10.1038/s41386-025-02192-2 (PMC12603268; doi:10.1038/s41386-025-02192-2)

Supplemental Figures

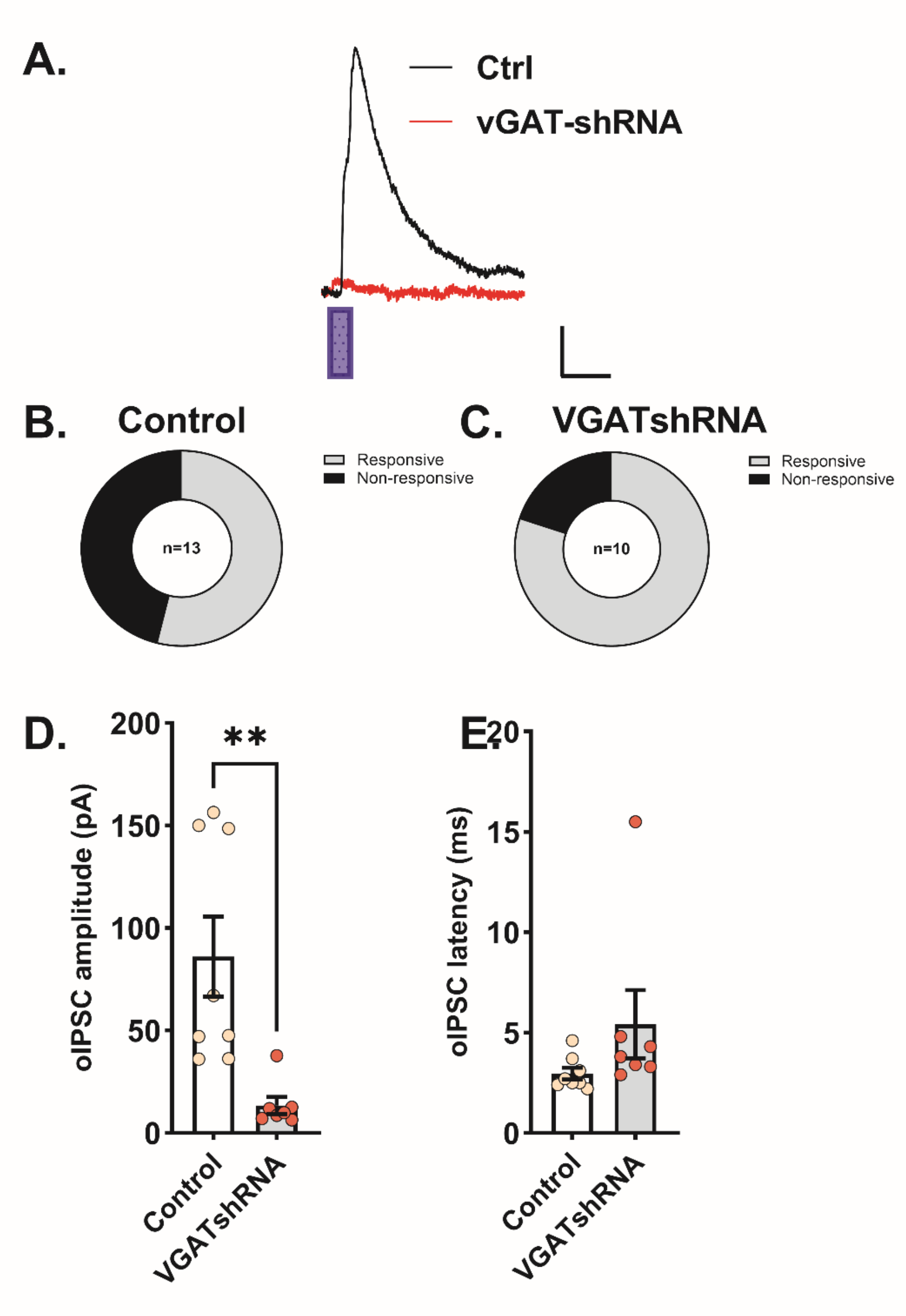

Supplemental Figure 1: Short hairpin RNA interference for the vesicular GABA transporter (shvGAT) reduces GABA release from ventral BNST CRF-expressing neurons

a. Representative Traces from control and vGATshRNA animals showing optical evoked release
b. Percentage of light-responsive non-fluorescent neurons in the BNST of male mice (n=2) injected with the control virus with a scrambled sequence
c. Percentage of light-responsive non-fluorescent neurons in the BNST of male mice (n=2) injected with the shvGAT virus. The percentages of responsive neurons were not different between virus conditions. Fisher's exact test p=0.4
d. The shvGAT virus reduced optically evoked inhibitory post-synaptic current amplitude compared with the scramble controls. t(7.6) = 3.6, p=0.007
e. There were no differences in latency for optically evoked inhibitory post synaptic currents between viral conditions t(13) = 1.5, p=0.15

MALE – SUCROSE - shRNA

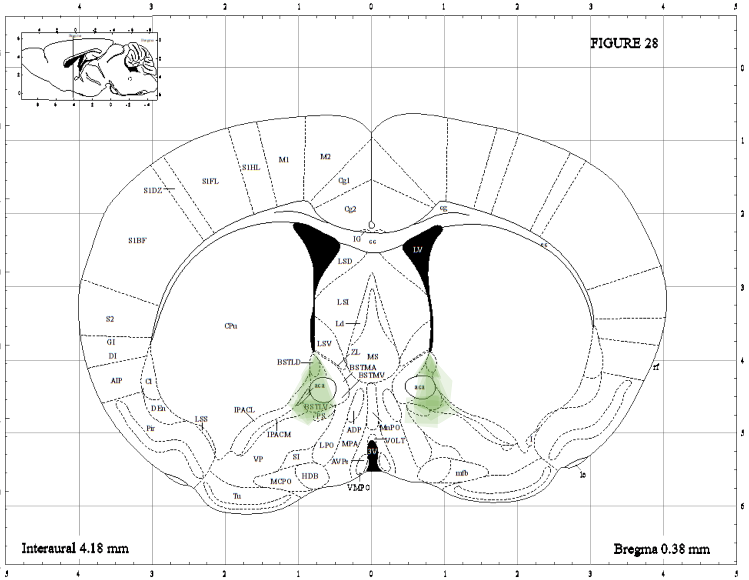

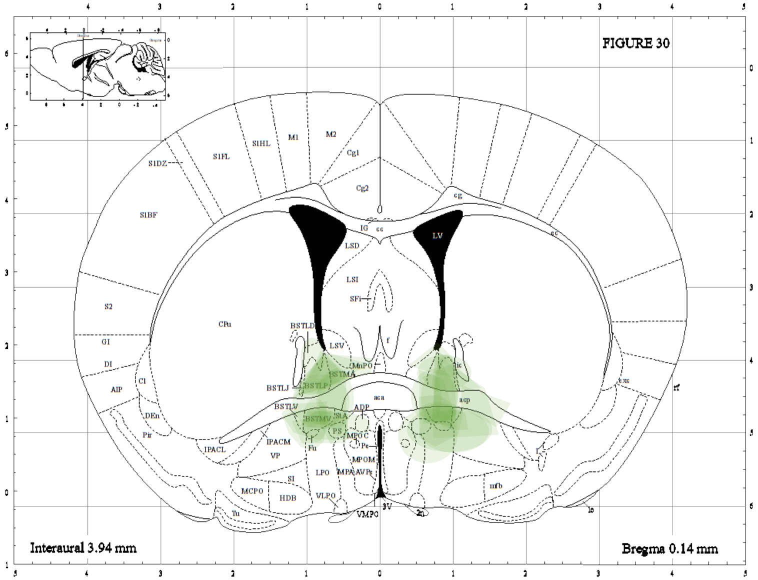

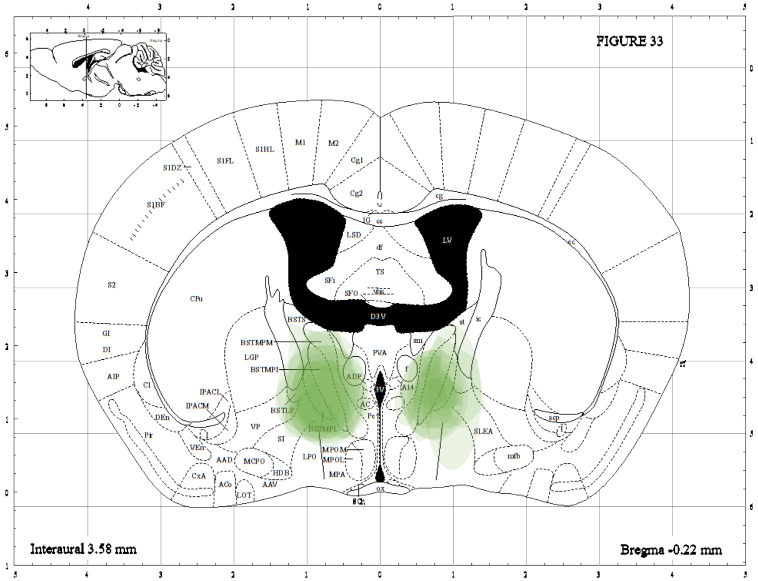

MALE – SUCROSE - Scramble

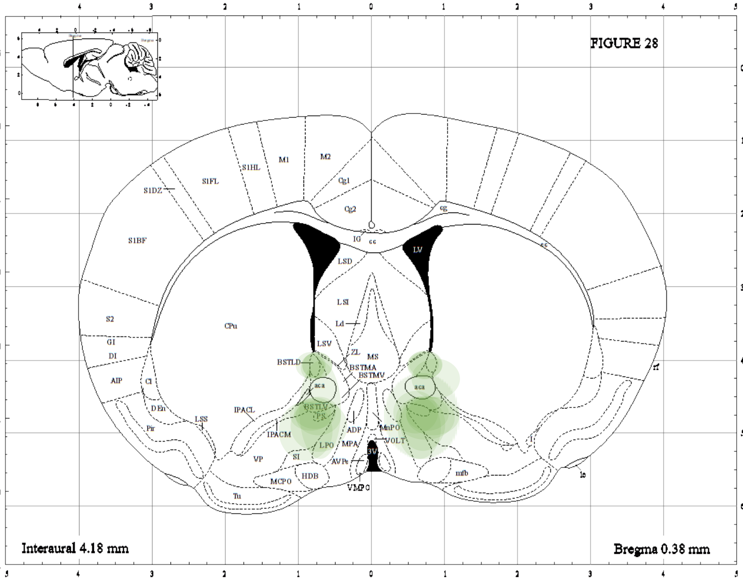

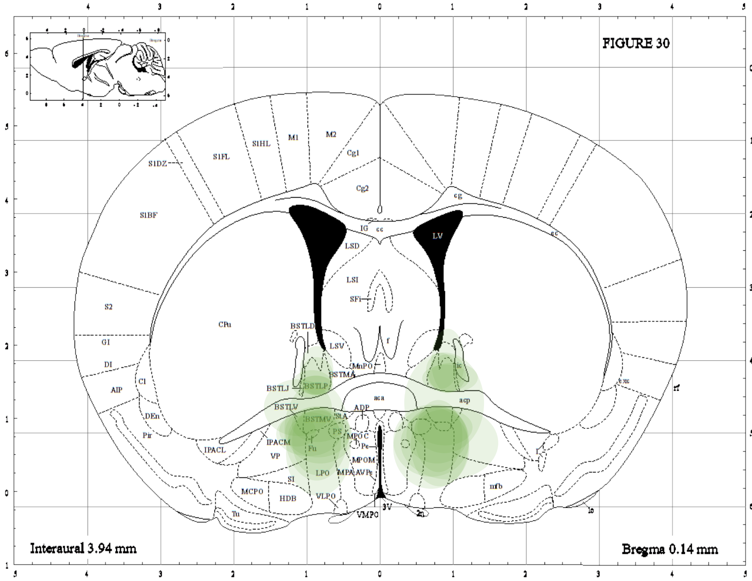

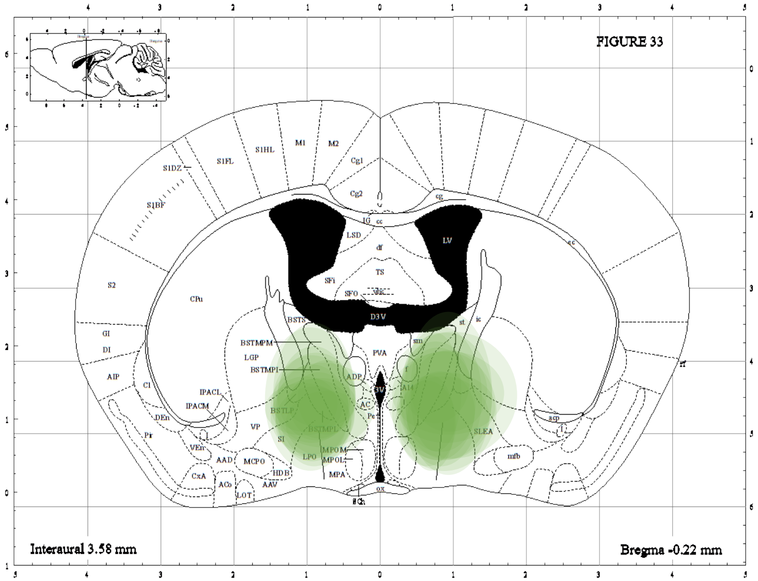

FEMALE – SUCROSE - shRNA

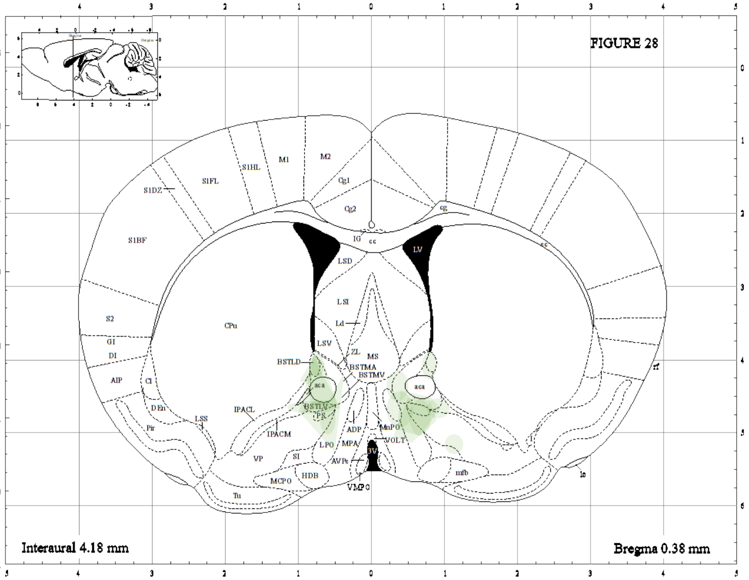

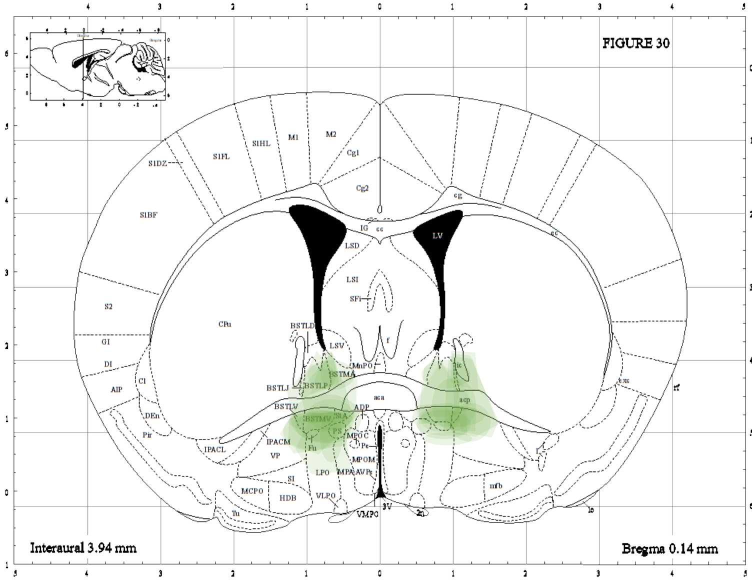

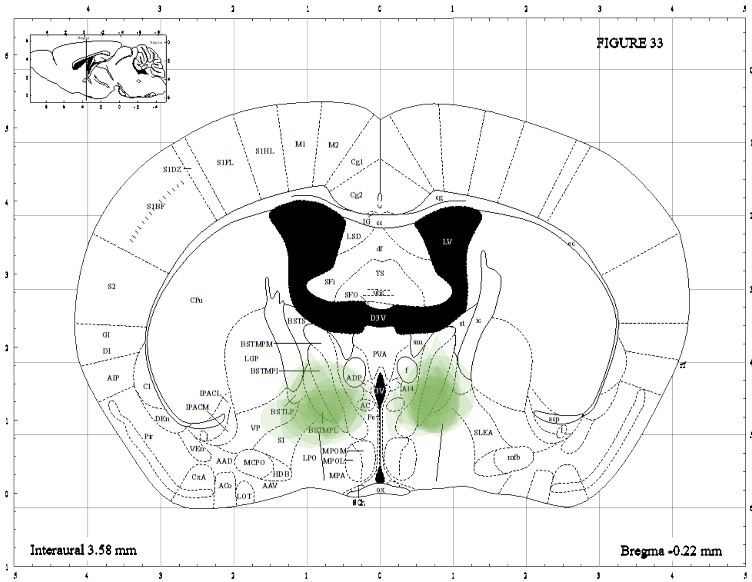

FEMALE – SUCROSE - Scramble

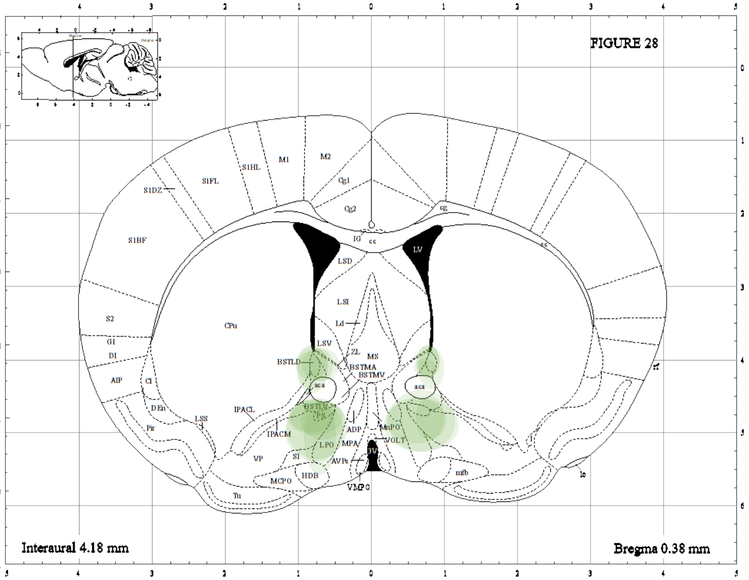

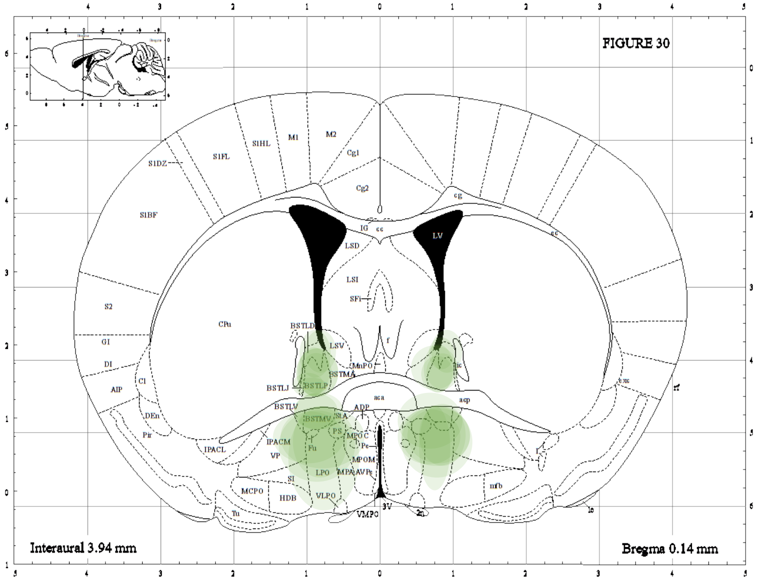

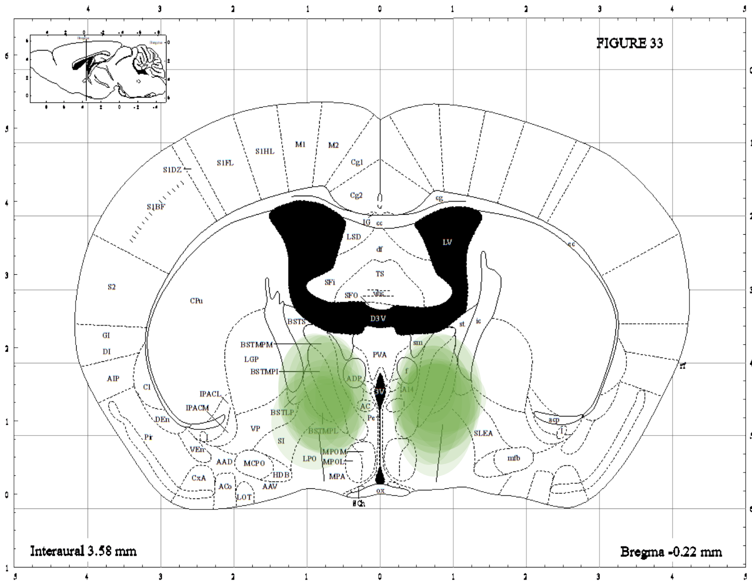

MALE – ALCOHOL - shRNA

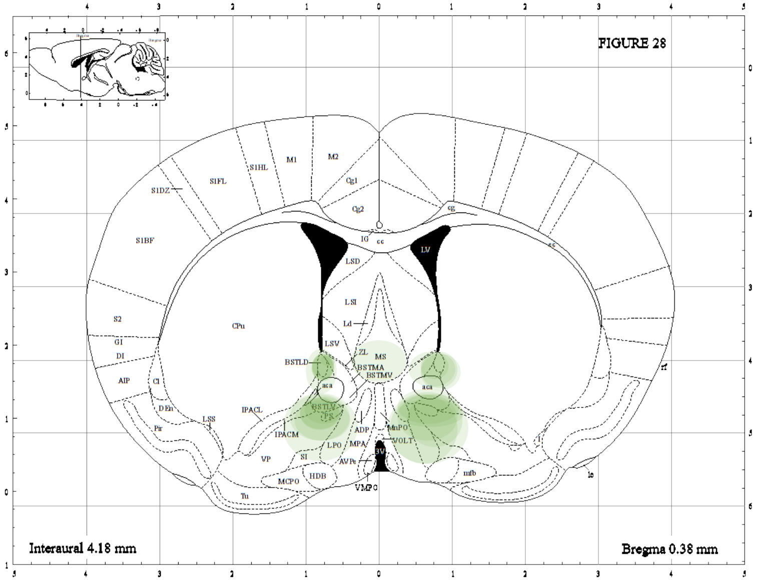

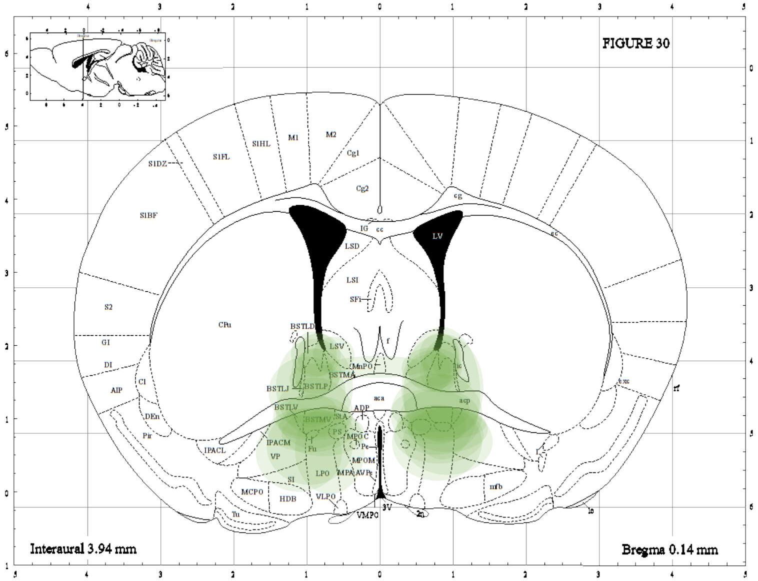

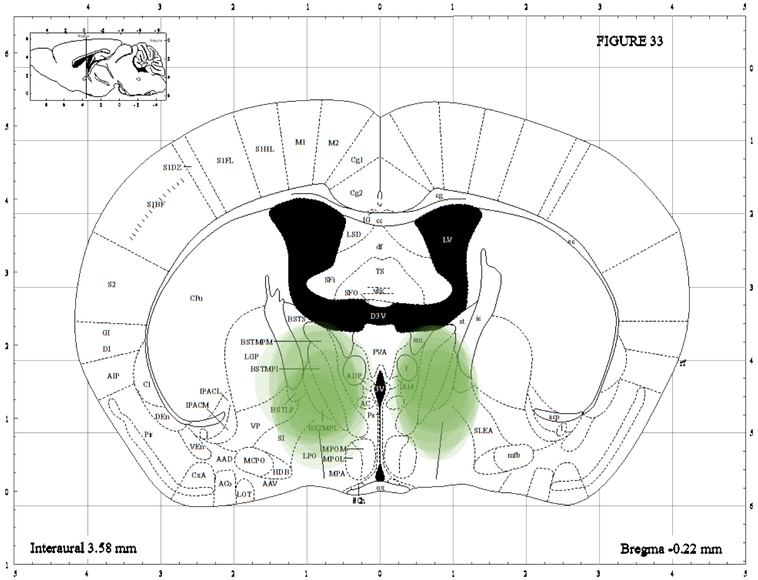

MALE – ALCOHOL - Scramble

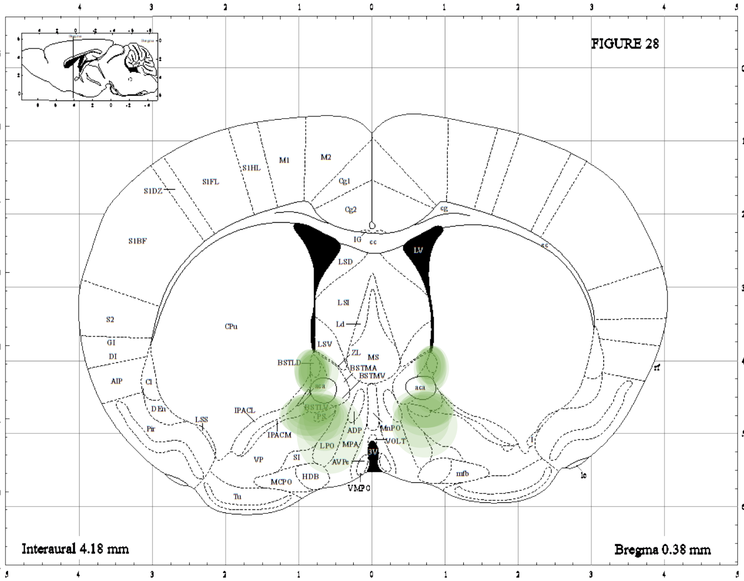

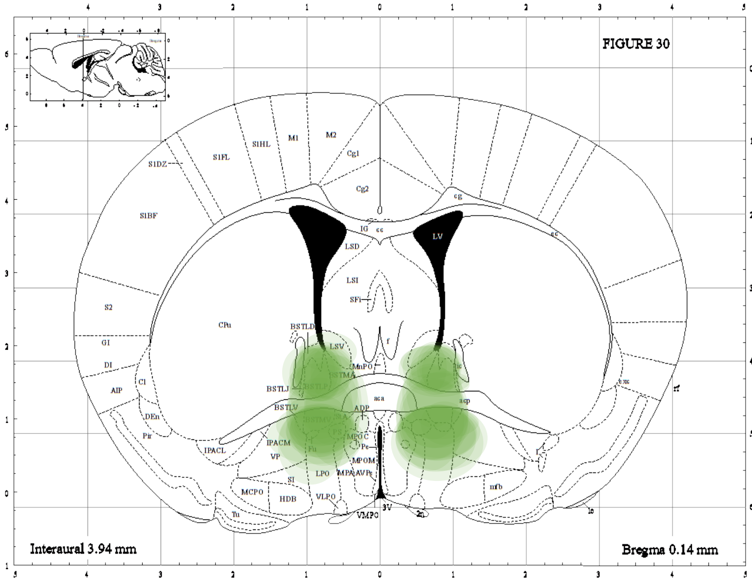

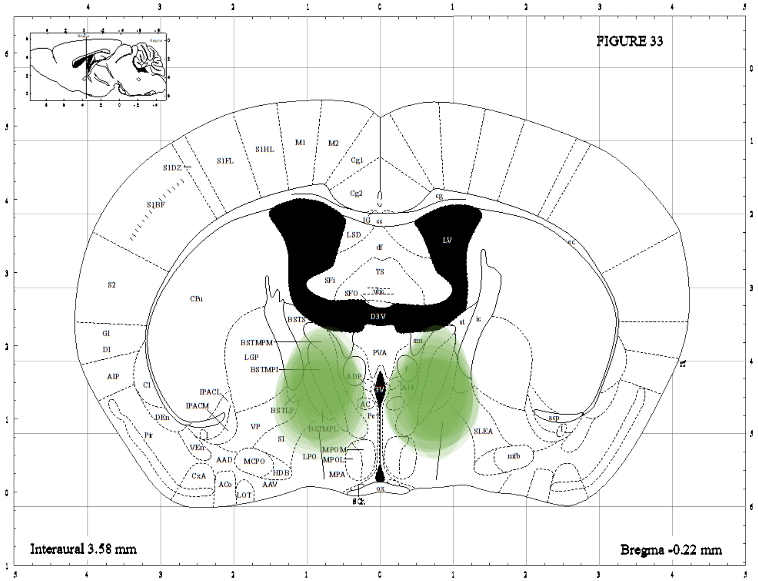

FEMALE – ALCOHOL - shRNA

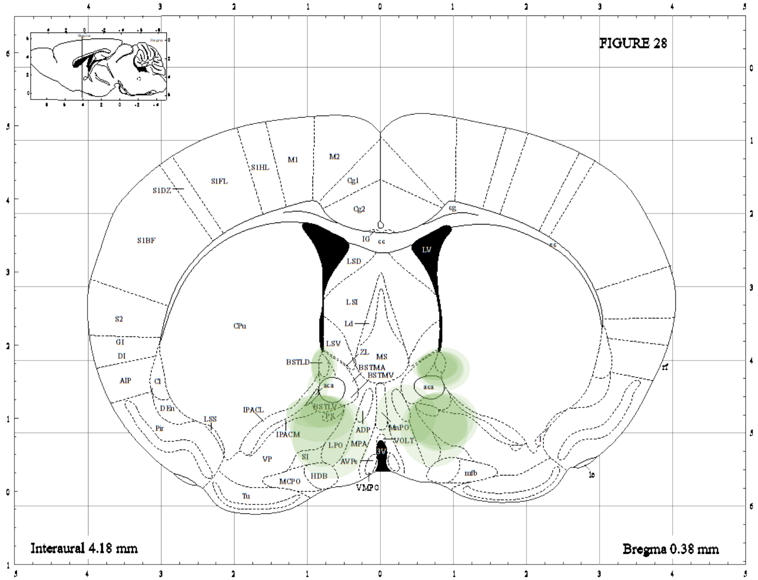

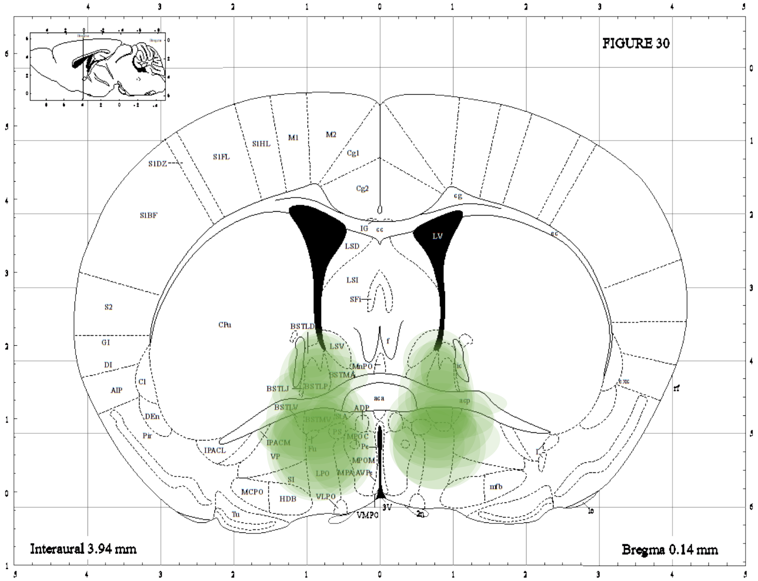

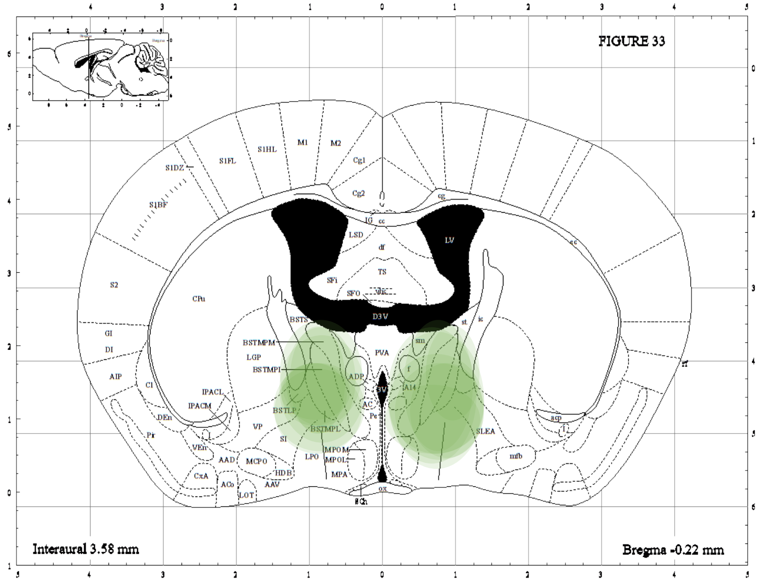

FEMALE – ALCOHOL - Scramble

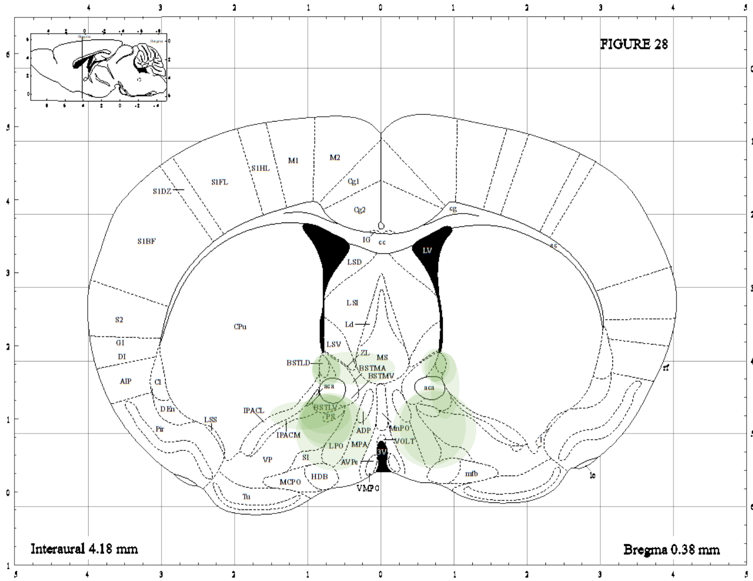

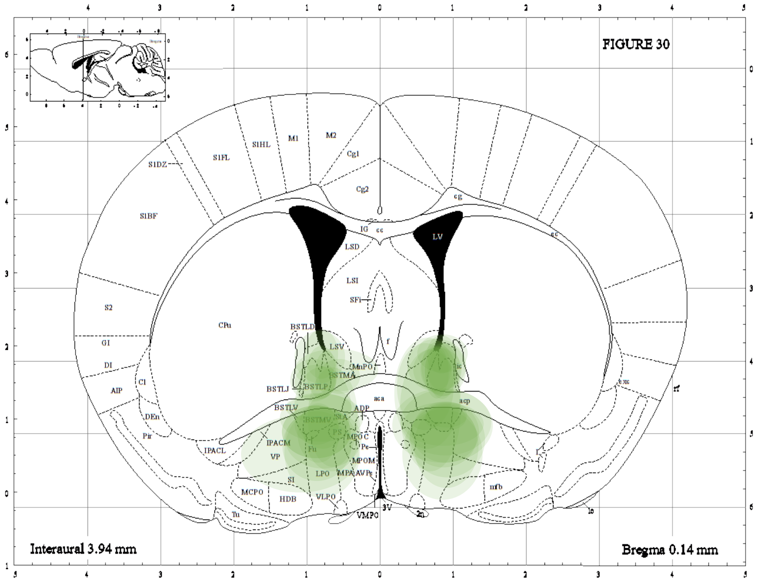

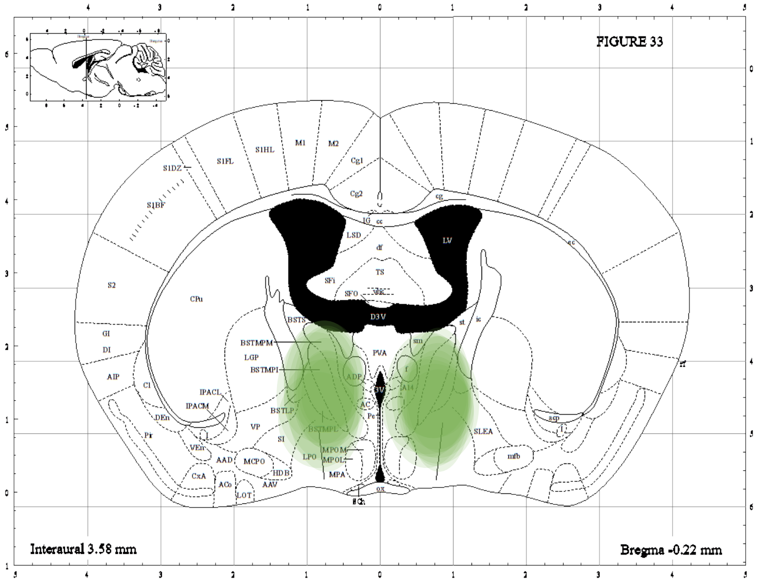

MALE – Sucrose - GFP

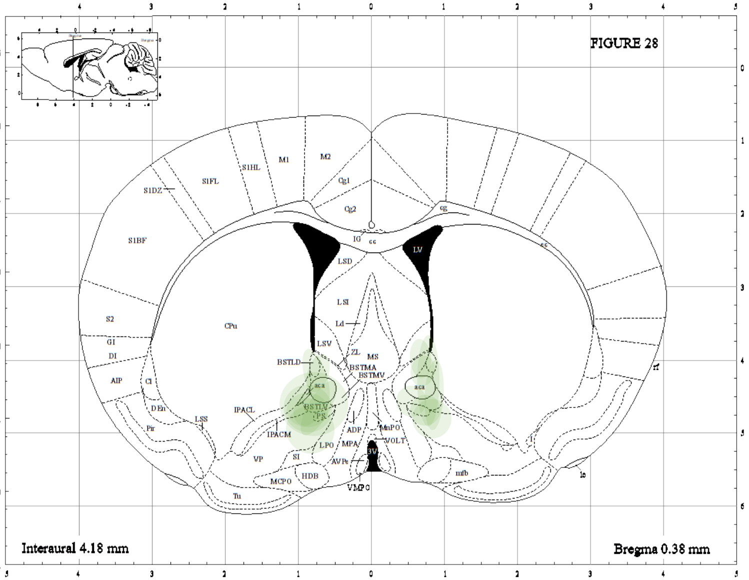

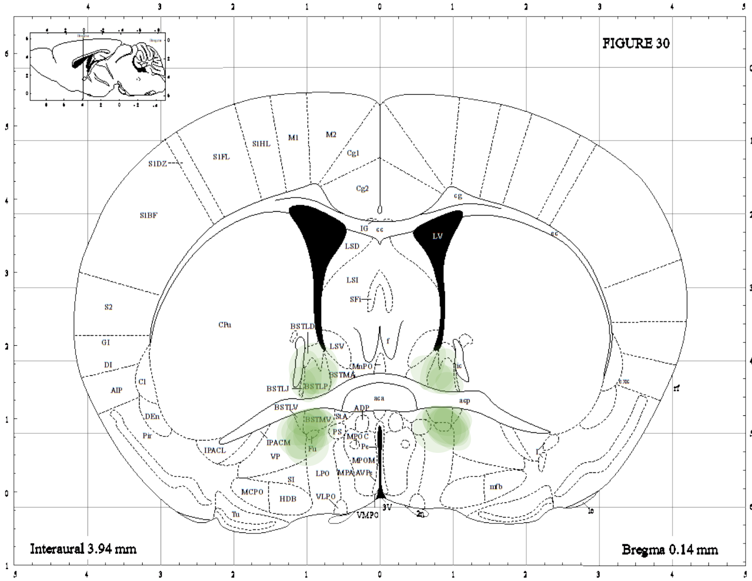

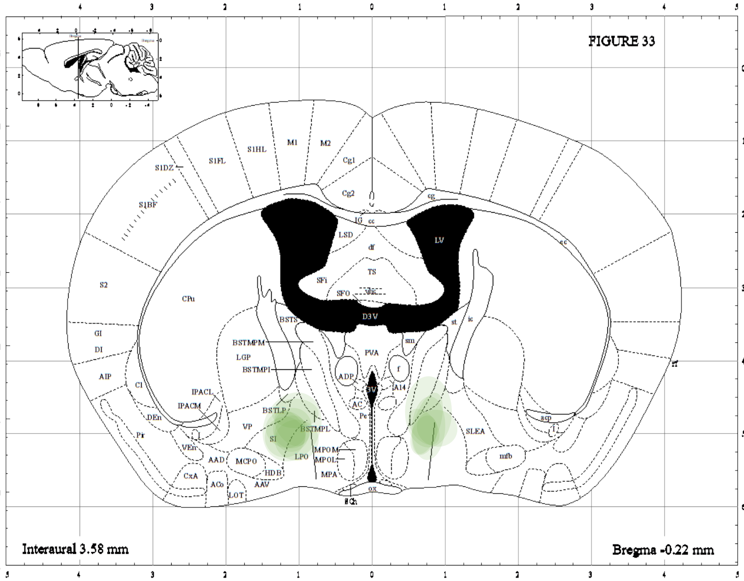

MALE – Sucrose - Cre

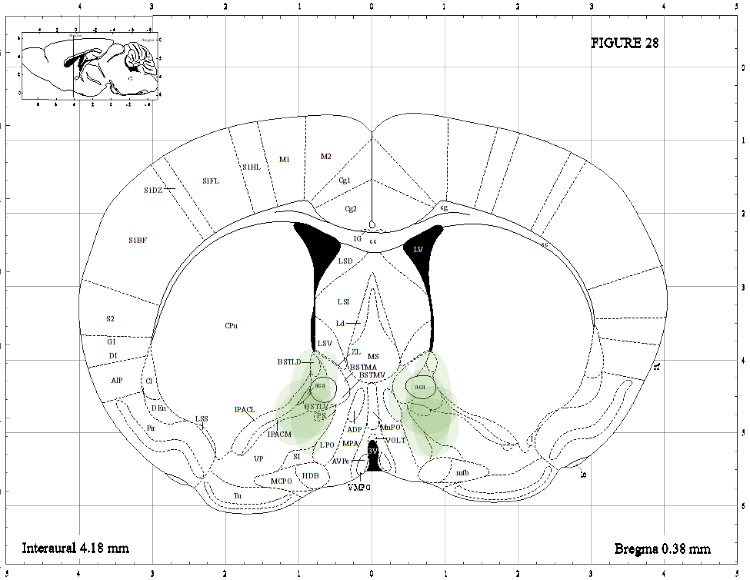

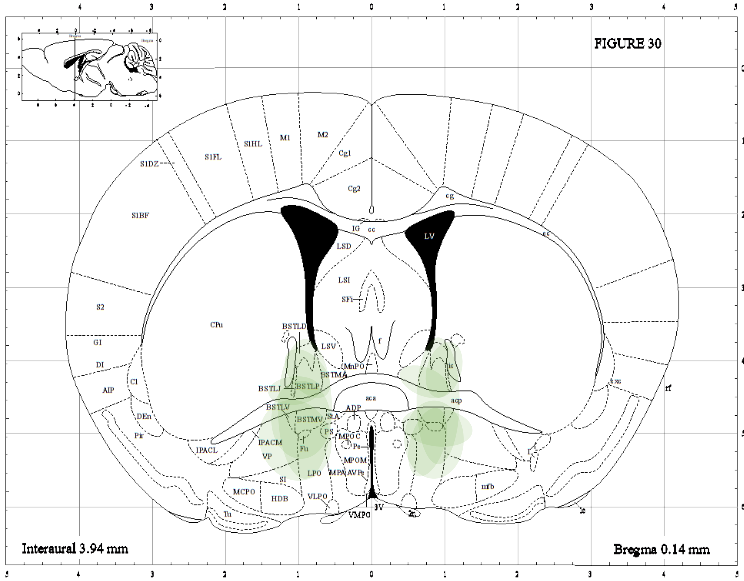

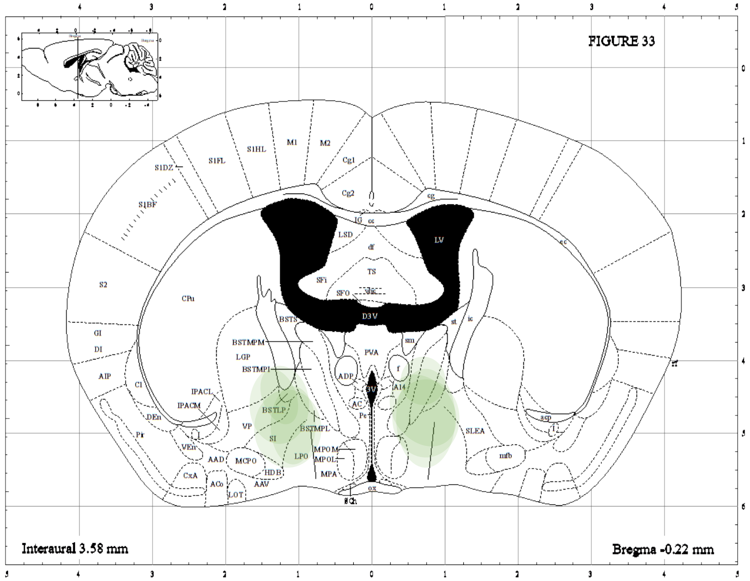

FEMALE – Sucrose - GFP

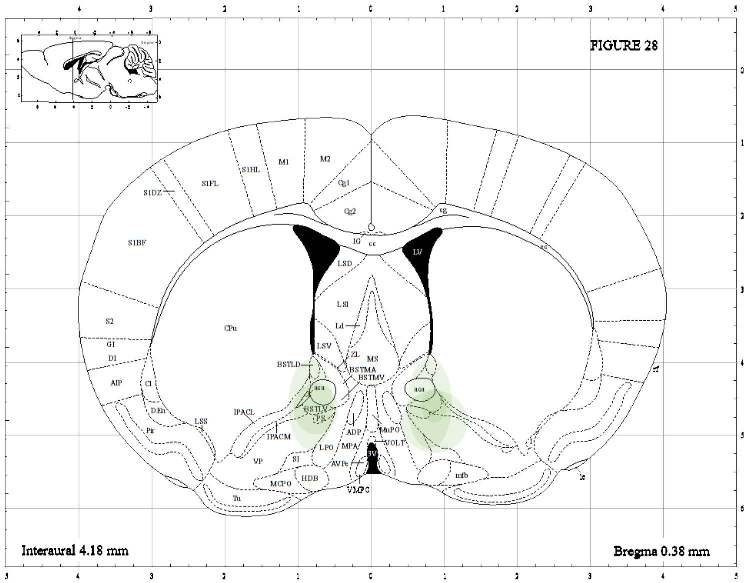

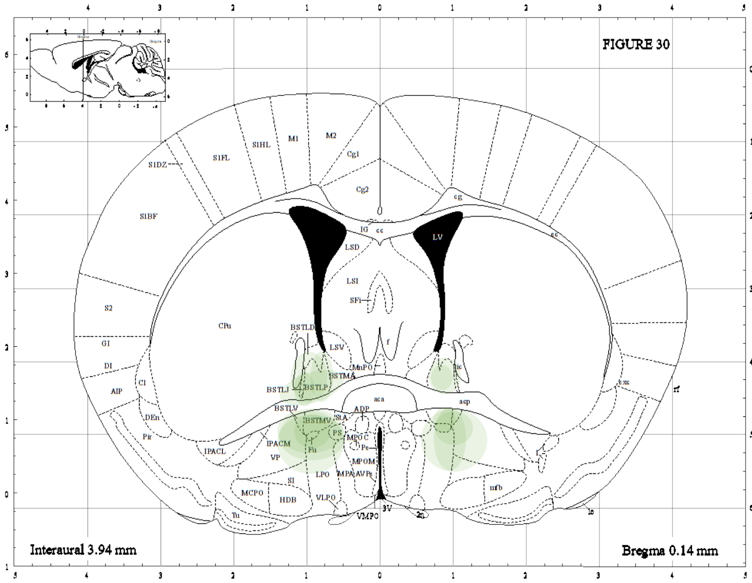

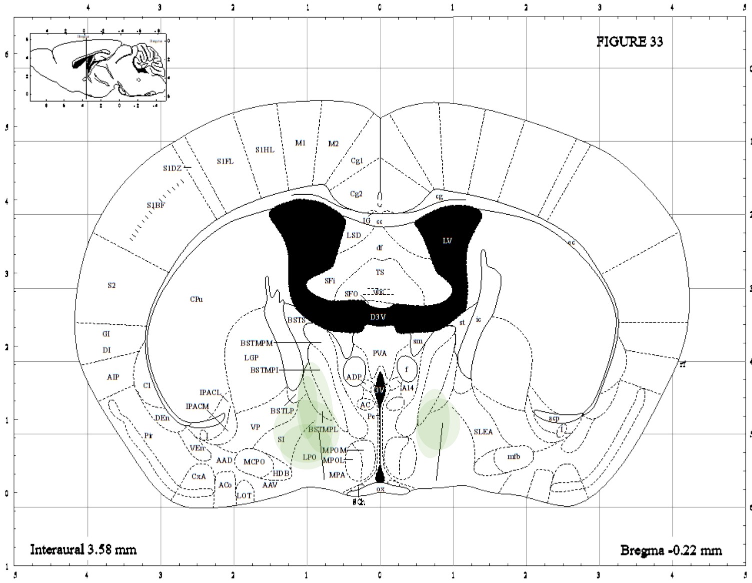

FEMALE – Sucrose - Cre

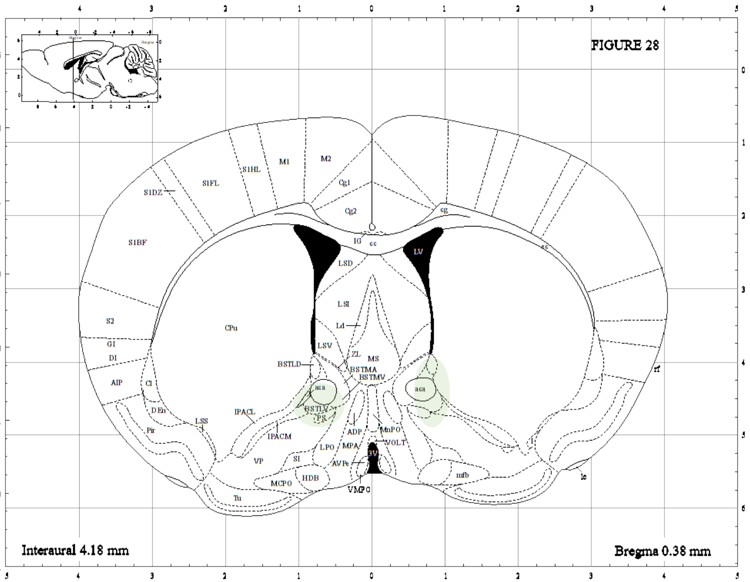

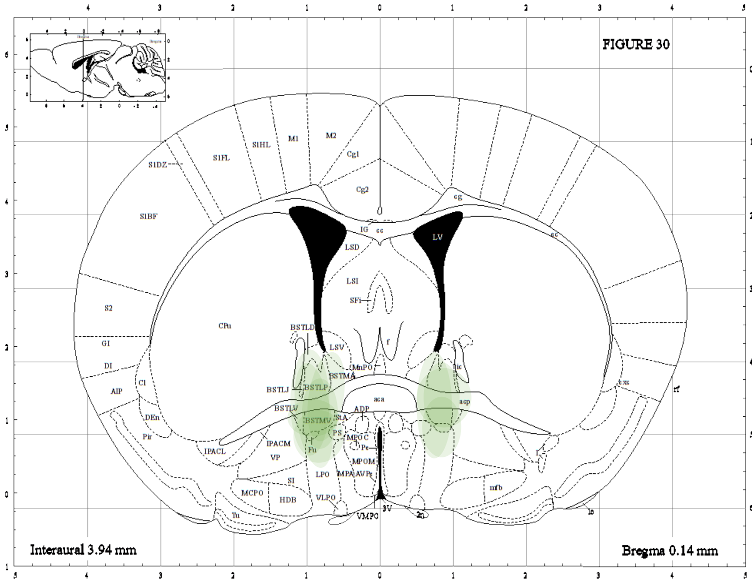

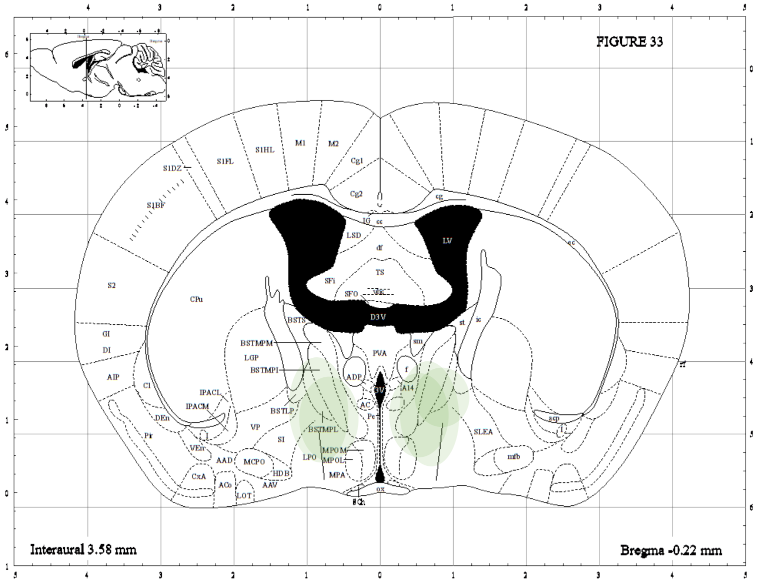

MALE – Alcohol - GFP

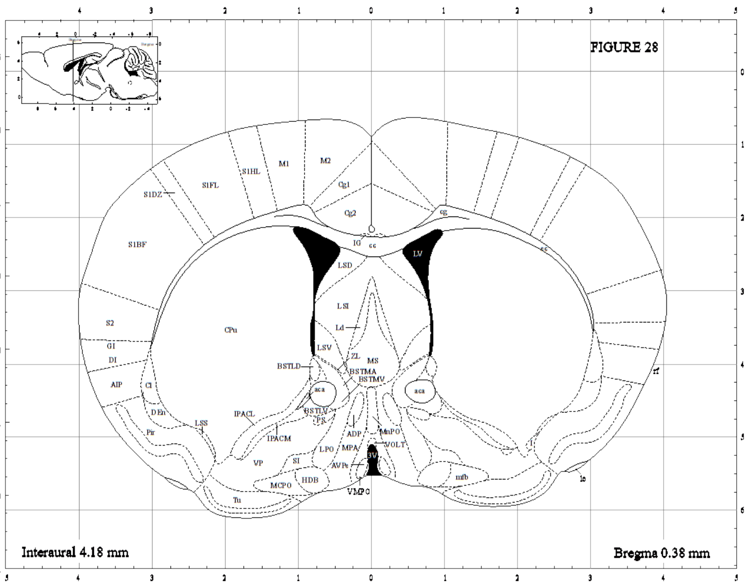

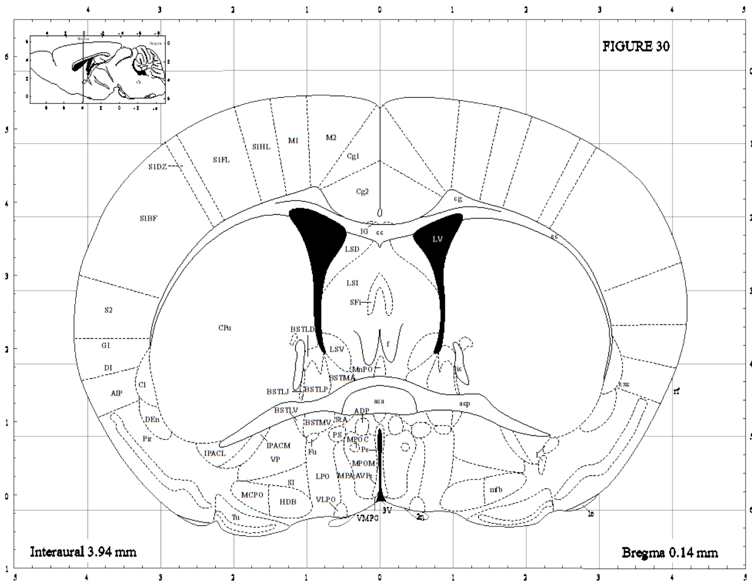

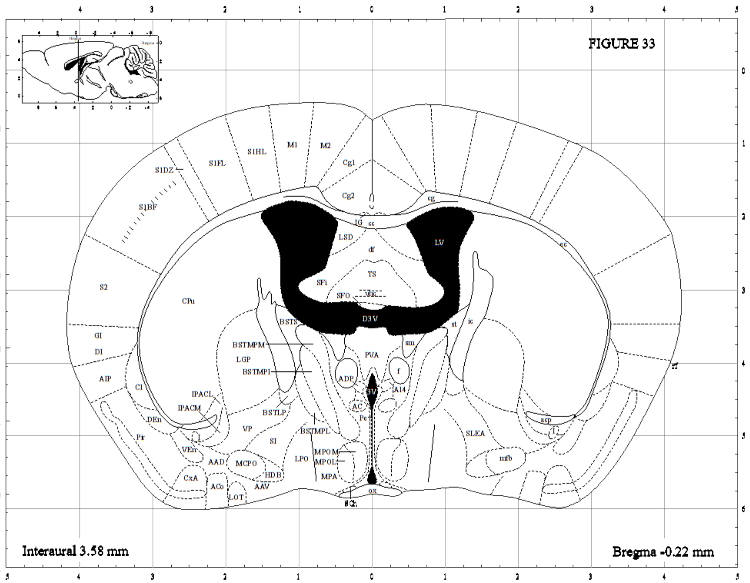

MALE – Alcohol - Cre

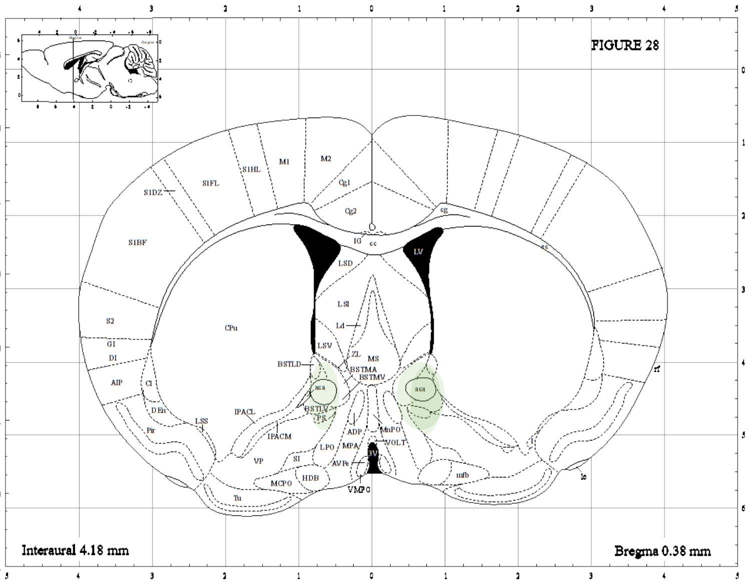

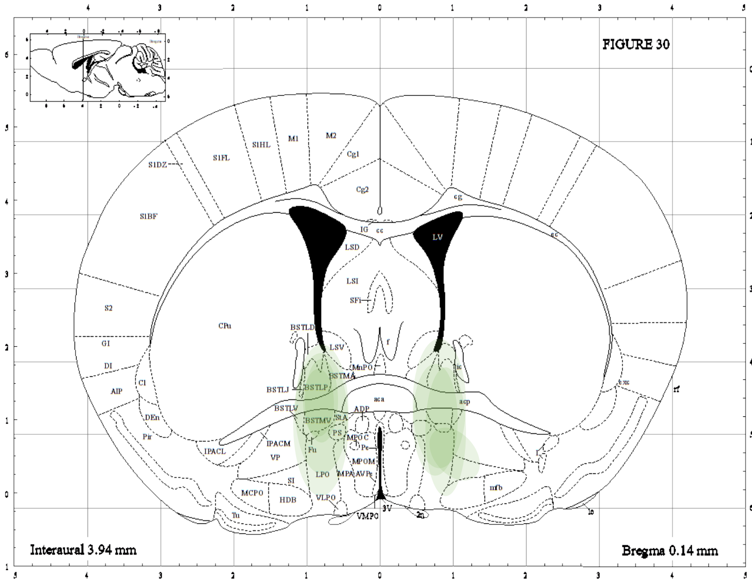

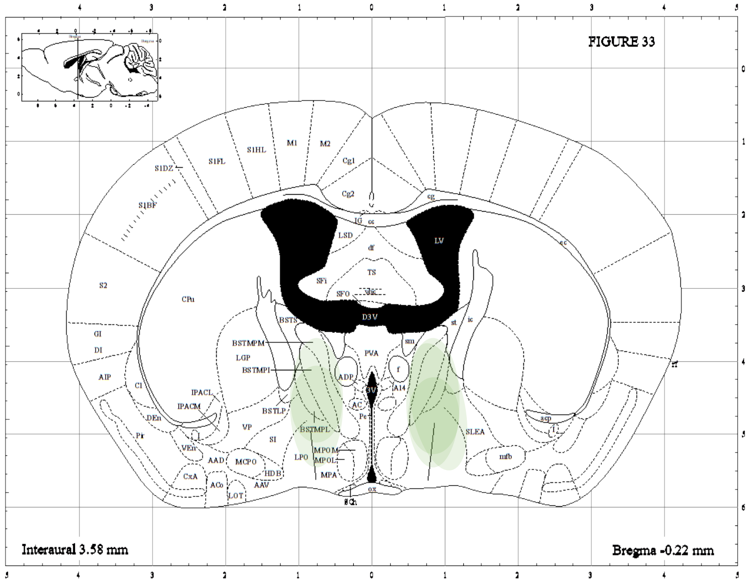

FEMALE – Alcohol - GFP

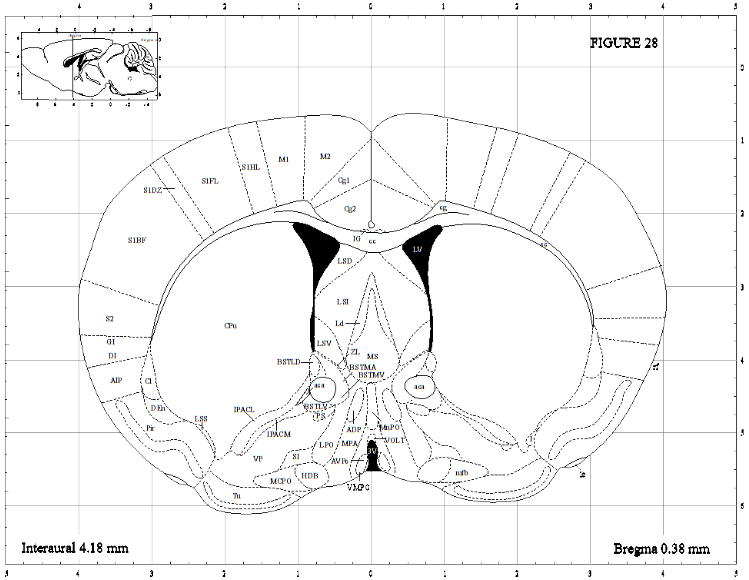

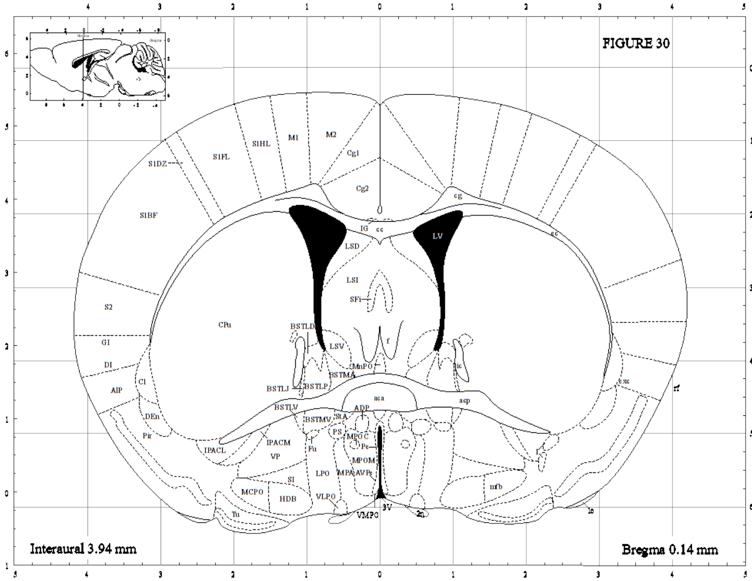

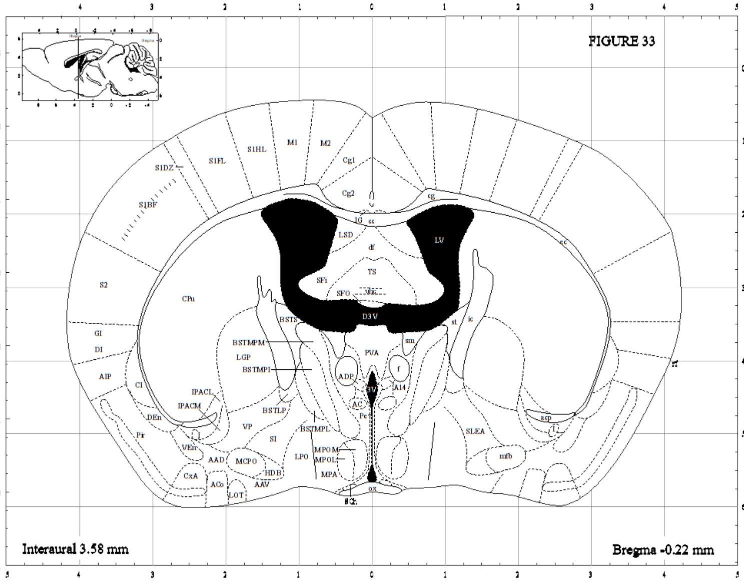

FEMALE – Alcohol - Cre

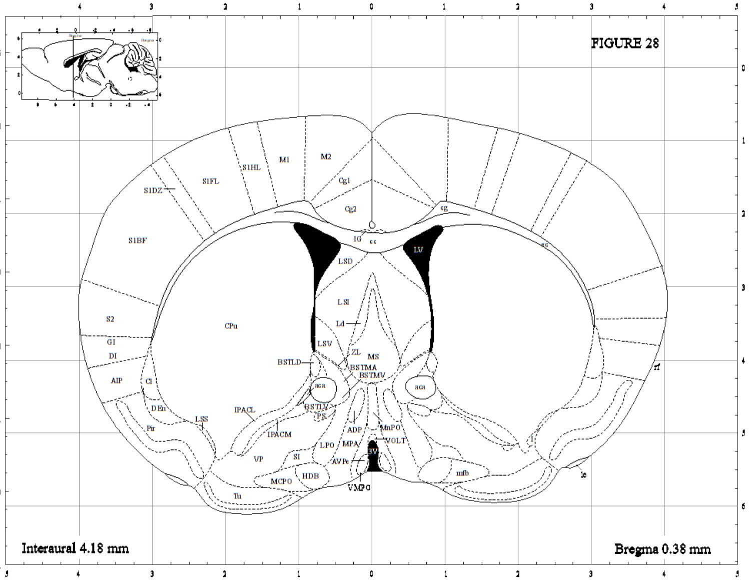

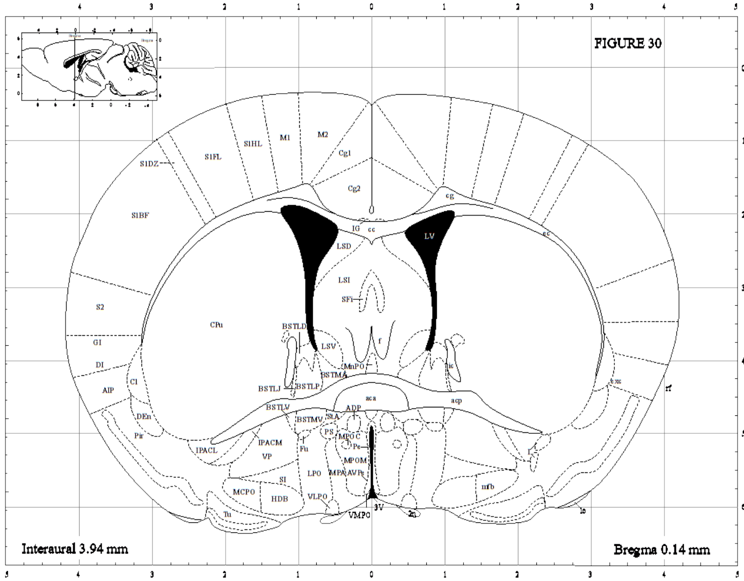

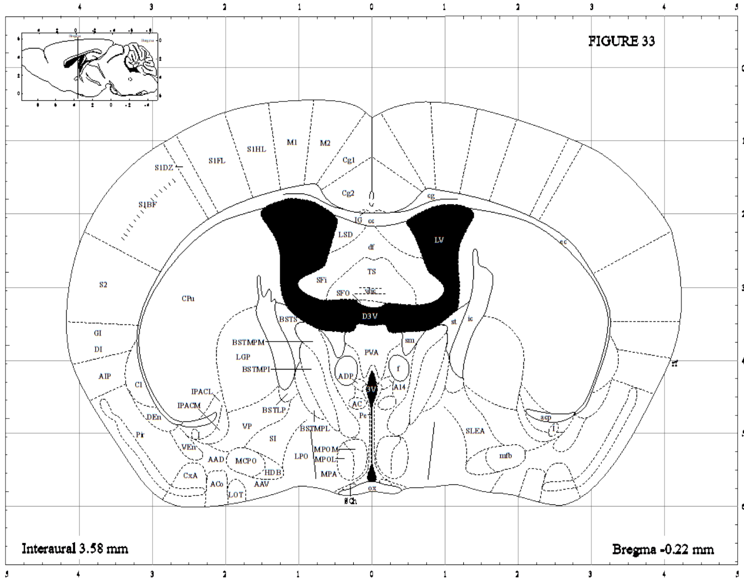

Supplement: Supplementary file 1 — Supplemental Data [file 41386_2025_2192_MOESM1_ESM.pdf]
